# A perspective: neuraxial therapeutics in pain management: now and future

**DOI:** 10.3389/fpain.2024.1505019

**Published:** 2024-12-10

**Authors:** Jose DeAndres, Anthony H. Dickenson, Salim Hayek, Andreas Linninger, Tony L. Yaksh

**Affiliations:** ^1^ Department of Anesthesia, Valencia University School of Medicine, Valencia, Spain; ^2^ Departments of Neuroscience, Physiology and Pharmacology, University College London, London, United Kingdom; ^3^ Anesthesiology Institute, Cleveland Clinic, Cleveland, OH, United States; ^4^ Biomedical Engineering and Neurosurgery, University of Illinois, Chicago, IL, United States; ^5^ Anesthesiology, University of California, San Diego, CA, United States

**Keywords:** neuraxial therapeutics, pain management, neuraxial delivery, therapeutic pain intervention, neuraxial targets, spinal drug delivery

## Abstract

The neuraxial delivery of drugs for the management of pain and other spinal pathologies is widely employed and is the subject of a large volume of ongoing research with several thousand papers appearing in the past 5 years alone on neuraxial delivery. Several learned texts have been recently published. A number of considerations have contributed to this widespread interest in the development of the use of neuraxial therapeutics to manage pain. In the following section, major topics relevant to spinal encoding and in the use of neuraxial therapeutics are considered by the Frontiers in Pain Research editors of the research topic: “*Neuraxial Therapeutics in Pain Management: Now and Future”*. This paper seeks to serve as a perspective to encourage the submission of manuscripts reflecting research in this exciting area.

## 1 Why neuraxial delivery?

Three condition may justify neuraxial delivery as a therapeutic intervention (1) The therapeutic target, in this case complex aspects of the pain experience, lies at the spinal level, as reviewed below. (2) The drug can reach the spinal parenchyma but achieves effective concentrations at doses which have adverse systemic effects (opiate, alpha2 agonists); and (3) the drug has no target access because of the blood brain barrier (ziconotide, viral transfection platforms). Under these common conditions (1–4), neuraxial delivery can be appreciated to have an important role in pain management.

### 1.1 Related research topics

• Historical commentary on the evolution of neuraxial therapeutics in. research and clinical practice
• Risk-benefit consideration governing neuraxial therapeutic delivery.
• Cost points for the use of neuraxial delivery in acute and chronic pain states.

## 2 The pivotal role played by spinal encoding complexities of a pain phenotype

This focus on neuraxial interventions in pain reflects the pivotal role played by the dorsal root ganglion (DRG) and the dorsal horn circuitry in many aspects of the pain states generated by tissue and nerve injury from the encoding and decoding of the content of the line labeled afferent message, to primary and secondary hyperpathia, to the anomalous events that result in pain secondary to activation of low threshold mechanoreceptors, and to the persistent presence of pain after resolution of the initiating injury leading to a chronic pain phenotype. Of equal importance, we appreciate that the pain state is composed not only of encoding that define the sensory-discriminative properties of the initiating stimulus, but directly contribute to input driving higher order functions that underlie the affective aspects of the pain stimulus which we consider relevant to the intense emotional components of pain (5–8). The efficacy of ventrolateral cordotomies in managing the crossed pain state was early evidence that such ascending information played a pivotal role in the pain experiences (9, 10). In addition to the ascending projections which exhibit somatotopy and projections into the somatosensory cortex, other projections reach the old limbic forebrain, long associated with affective aspects of behavior and to regions such as the hypothalamus, amygdala, and hippocampus reflecting connectivity involved in trophic regulation, learning and memory (11, 12). Thus, the evident therapeutic efficacy of neuraxial interventions in managing pain states generated by acute and chronic conditions secondary to tissue and nerve injury has pointed to the evident role that such control of the spinofugal message has profound effects not only on pain intensity but the emotive and autonomic aspects of the pain experience. It appears that these afferent-spinofugal linkages have a discrete pharmacology, suggesting specific targeting by spinal therapeutics of the components of pain information they process (12–14).

### 2.1 Related research topics

• Dorsal horn linkages regulating input-output function of distinct populations of spinofugal axons.
• Role of identified spinofugal projections into brainstem, diencephalon and forebrain in the encoding of nociceptive stimuli and their higher order projections to areas associated with somatic, autonomic or affective behavioral components.
• Supraspinal linkages activating. descending pathways that up- or down-regulate nociceptive signaling, e.g., reflecting hyper- or hypo-algesia, respectively.

## 3 Neuraxial targets

Many states of severe pain and spasticity reflect processes regulated at the spinal cord. Preclinical models for neuraxial delivery, in conjunction with sophisticated behavioral assessments strategies for pain phenotypes, has permitted characterization of the role of pharmacologically defined targets on nociceptive processing across species (15). These study models have permitted systematic characterization of virtually all current therapeutics regulating pain (such as opiates and alpha 2 agonists; NaV 1.7, CaV2.2 channel blockers) (15–17) or spasticity (GABA A agonism) (18) acting at the spinal level to regulate this processing (19–21).

Earlier studies emphasized the role of a myriad of neuropeptides in the spinal cord and in the dorsal root ganglion (22), forming local circuits though eponymous receptors on second order interneurons and projection neurons for cholecystoknin, gastrin-releasing peptide, substance P, Neurotensin, neuropeptide FF (23). The role of these local circuits in nociceptive processing has become increasingly appreciated as potential targets (24). As an example, local CCK circuits have been identified (25). Blocking spinal CCK receptors prevents allodynic states (26–28) and serves to prevent up regulation of proinflammatory signaling (29). Aside from neurons, DRG macrophages and satellite cells and dorsal horn astrocytes and microglia play a crucial role in regulating the excitability of the dorsal horn input-output function (30–33). There is a growing appreciation of the role of innate and adaptive immunity in mediating pain phenotypes evoked by circulating immune complexes (34, 35) and the ability of these products to reach the DRG after intrathecal and systemic delivery though its unique ganglion-blood barrier (36) points to the evolution of neuraxial pain biology that may be directly addressed by neuraxial delivery. Based on behavioral studies with neuraxial therapeutics, regulation of this diverse neuraxial biology has relevance to pain phenotypes identified as nociceptive, neuropathic and nociplastic (37).

### 3.1 Related research topics

• Advances in development of neuraxial delivery models to permit study of the effects of acute and chronic delivery of neuraxially targeted therapeutics in the animal.
• Specific assessment of role of neuraxial targets in regulating complex pain behaviors and phenotypes.
• Changes in higher order function as defined by non-invasive imaging and electrophysiology produced by neuraxial therapeutics.

## 4 Therapeutic delivery platforms and formulations for neuraxial delivery

Neuraxial delivery commonly employs small molecules delivered in a water based vehicle. The pharmacokinetics of such formulations has been suitable for short to moderately long lasting therapeutic effects. Maintenance of drug action for longer periods has required multiple deliveries or infusions though implanted devices. The current area of therapeutic platforms has been markedly broadened with the development of transfection motifs such as antisense oligonucleotides and viral transfection platforms or targeted neurotoxins, which permits the targeting of neuraxial specific genes to increase or decrease protein expression to address neurological pathologies such as in pain, spasticity or neurodegenerative disorders (38–43). These platforms provide an opportunity to address the cause of pathology and thus qualify as disease modifying. Transfection of transducer protein activated by novel designer ligand [DREADD: (44)], light [optogenetics: (45)] or ultrasound [sonogenetics: (46)] permits creation of novel interventions to regulate nerve, DRG and spinal function. Future development of these approaches will witness the ability to regulate the duration of the induced alterations by making the changes conditional and subject to on/off regulation. Such capabilities promise to robustly expand the use of these neuraxial modalities to meet the clinical pain phenotype (e.g., acute: day to weeks- post surgical; semi chronic: weeks to months—trauma, burn; and, chronic: non reversible- terminal, genetic, degenerative pathologies).

### 4.1 Related research topics

• Delivery formulations which alter bioavailability and pharmacokinetics of neuraxial therapeutics.
• Targeting platforms that alter for varying intervals neuraxial processing though transfection and toxins motifs and/or are subject to regulation (e.g., conditional Knock in/Knock outs).

## 5 Neuraxial CSF/parenchyma drug distribution

The fluid filled intrathecal space is confined within a moderately compliant dural sac which undergoes limited, periodic, oscillatory-movement. This movement is driven by pressure gradients that arise from: (i) changes in the non-compliant intracranial blood volume, forcing caudal movement of ventricular cerebrospinal fluid (CSF) (46–52); (ii) compression of the spinal thecal sac by fluctuations in blood volume in the perispinal venous plexus (Batson's plexus) induced by inhalation and expiration (53–56) and (iii) arterial pulsations along the neuraxis (e.g., via the intercostal and radicular arteries (57, 58).

In accord with these properties, intrathecal injectates delivered at low rates or in small volumes display minimal pericatheter or rostrocaudal redistribution (59–63). As will be noted in the following section, these low flow patterns and their associated gradient are associated with restricted redistribution of the injected therapeutic and delivery strategies to enhance distribution are of interest. An additional variable regarding CSF-solute movement of the drug following intrathecal delivery is into the parenchyma or to the DRG. Current thinking has emphasized that the physicochemical properties of the molecule (lipid partition coefficient, molecular weight, etc.) serve to define the facility of moving from the CSF into the parenchyma. In this regard, the pia represents a surprisingly substantial barrier for diffusion of larger molecules and particle, as with viral transfection (64, 65). Once in the parenchyma, molecules exhibit diffusion properties defined by physical characteristics of the molecule, including molecular weight and lipid solubility (66, 67). Of note, after IT delivery there is substantial movement of even large molecules or particles into the dorsal root ganglion. This selectivity is widely appreciated for the ability of intrathecal adenoviruses to result in DRG transfection (17).

### 5.1 Related research topics

• Assessment of the intrinsic flow patterns in the extracranial neuraxial space and physiological factors (respiratory/cardiovascular) altering that flow pattern.
• Characterization of factors such as respiratory and cardiac cycles and changes in thoracic/abdominal pressures influencing CSF flow patterns.
• Consideration of factors governing distribution of intrathecal/epidural molecules to and into the CSF, meninges, parenchyma and DRG (e.g., lipid partition coefficient, molecular weight, formulation/encapsulation)
• Characterization of route by which intrathecal molecules/particles reach the DRG.

## 6 Interventions to regulate spinal drug distribution

As reviewed above, movement of cerebrospinal fluid within the extracranial neuraxial, while present, is restricted. In accord with these properties, intrathecal injectates delivered in low volumes and low rates often display minimal pericatheter or rostrocaudal redistribution (59–63). These injected solutions typically show a gradient away from the site of focal drug delivery with only modest lateral movement (20, 59, 68, 69). Here, issues of interest relate to increasing local spread of the injectate away from the injection or infusion sites, reducing local tissue exposure and (ii) promoting, as required, the degree of rostrocaudal spread.

(i) Pericatheter redistribution. As reviewed below, neuraxial formulations may employ high concentrations. The absence of a robust redistribution away from the catheter means that local tissue is exposed to that concentration, enhancing the likelihood of local toxicity (69). Strategies to increase lateral movement involve increasing exit velocity of the solute from the catheter/needle). This is practically accomplished by increasing the delivery rate (e.g., bolus vs. infusion) and increasing exit resistance from the catheter (20, 21, 69).
(ii) Rostrocaudal distribution. Spinal nociceptive processing from a segmental input is not limited to a single spinal segment. Afferent traffic from a single nerve root may communicate with spinal levels as much as 5–10 spinal segments distant (70). Accordingly, a spinal drug, which is believed to act on the afferent terminal, such as a mu opioid or alpha 2 adrenergic (15), must reach spinal levels spanning this distance at effective doses to prove efficacious. The issue of achieving extended rostrocaudal redistribution is particularly important for neuraxial pathologies (infection/cancer) or neurodegenerative processes (e.g., Somatomotor Atrophy/Amyotrophic Lateral Sclerosis) where the delivery of the therapeutic requires sacral to cervical distribution (71, 72). Not surprisingly, Increased rostrocaudal redistribution of the injected drug may be achieved by greater injection volumes or higher rates of infusion. *In vivo* studies (monkeys) show that acute high-volume injections of tracers can generate greater distribution across the neuraxis (73) as a result of (1): higher injection volume leading to a wider initial spread, followed by (i) rapid dispersion mediated by natural CSF pulsation of a given amplitude and frequency around spinal microanatomical features which give rise to geometry-induced mixing effects especially around nerve roots and trabeculae (74, 75). Lipophilic agents are typically cleared from the CSF into adjacent tissues and therefore show less rostrocaudal movement than non-diffusive tracers that are not taken up. Volume restrictions and effects upon neuraxial pressure are limiting factors in a volume based protocols (76) and higher rates of delivery from a point source may have limited effects upon rostral-caudal distribution due to the anatomical complexity of the intrathecal space (20). Further, the increased rostrocaudal effect is achieved at the cost of much higher doses of drug exposure at the site of injection with consequences as noted below. Alternate possibilities have focused on the catheter itself, using catheters with multiple high resistance exit sites, e.g., valves leading to increased exit velocities (20, 21) or multiple orifices (77).
(iii) *In vitro* modeling. Current work to model delivery parameters and their impact upon distribution has taken advantage of mathematical and physical models to predict the effects of formulation and delivery parameters to produce defined degrees of redistribution (74, 75, 78–81). These models can be dimensionally comparable to the anatomy and possess the physiologically relevant characteristics, including the sources of pressure gradients (vascular, respiratory) and the compliance of the intrathecal space as defined by the local arterial and venous vasculature and dural compliance). A 3D printed subject-specific deformable phantom models of the human central nervous system has been used to study intrathecal infusion under realistic physiological conditions (81). This model showed strong micro-mixing effects due to anatomical features in the spinal subarachnoid space which served as active drivers of intrathecal drug dispersion. The effect of infusion parameters (injection rate volume and solute dilution) and physiological factors (CSF volume, amplitude and frequency) can be quantified over an entire range of physiological ranges in human CNS. Moreover, the effective dispersion coefficient as a function of CSF properties (mainly amplitude and frequency) can be determined with optical techniques.

### 6.1 Related research topics

• Device (catheters, ports and pumps) design and functional properties promoting local redistribution of a neuraxial drug and the effects of such variables on therapeutic efficacy, efficiency and safety.
• Neuraxial delivery protocols to facilitate pericatheter and rostrocaudal injectate. distribution
• Role of injection formulation (baricity, encapsulation), volume and rate of delivery on distribution.
• Strategies to enhance supraspinal redistribution after extracranial neuraxial delivery.
• Development of models to assess and predict neuraxial distribution.

## 7 Clinical issues in the use of neuraxial therapeutics

Intrathecal delivery of therapeutics is used for the efficacious management of acute and chronic pain of a somatic or neuropathic origin (82). Advancing an agent for intrathecal drug delivery (IDD) through regulatory agencies is more challenging than for systemic applications. Thus, clinical implementation of intrathecal drugs has lagged. This slower development reflects safety concerns, patient population size, regulatory requirements, manufacturing and stability, cost of development and reimbursement potential (15). Hence, only two agents have gained regulatory approval for IDD for chronic pain: preservative-free morphine and N-type calcium channel blocker ziconotide. Intrathecal morphine displays varying degree of tolerance in animal models and in humans (83–85). Ziconotide has a narrow therapeutic window and significant neurocognitive side effects. Hence, practitioners have resorted to identifying clinical patient and drug factors that are important for optimizing IDD and have additionally used several off label intrathecal adjuvants, which while not approved for IDD, have become a standard of care in clinical practice (20). These therapeutics include other opioids (hydromorphone, fentanyl and sufentanil) as well as a local anesthetic (bupivacaine) and an alpha-2 adrenergic agonist (clonidine). For reasons outlined below (Neuraxial safety), the safety of agents used for neuraxial use in humans requires systematic preclinical assessment. These off label agents are employed with the rationale that their long use with little evidence of adverse events renders them acceptable. Clinical parameters that are favorable for minimizing intrathecal opioid dose escalation include older patient age (86) and minimal to no baseline systemic opioid prior to initiation of IDD (87, 88). Co-administering the local anesthetic bupivacaine from the outset of opioid IDD may blunt opioid dose escalation (89). For ziconotide, use of low doses and slow titration without other concomitant agents in the pump may prove valuable in effecting pain relief (90). Given the large number of management permutations and limited clinical data, consensus statements have been developed regarding best practices for patients suffering with cancer related pain and those with refractory chronic non-cancer pain (82). In general, and given limited CSF flow dynamics both rostrocaudally and circumferentially (see above), clinicians strive to place IDD catheters in the dorsal intrathecal space in close proximity to putative segmental targets. This may be more important for lipophilic agents than hydrophobic agents (68, 91). Nonetheless, significant challenges remain with efficacy and tolerability of commonly used agents highlighting the need for novel agents and improved delivery techniques.

### 7.1 Related research topics

• Appropriately powered and controlled clinical trials reflecting the effects of neuraxial therapeutics on the spectrum of pain states
• Clinical implementation of neuraxial therapeutics for chronic delivery using catheters accessed by subcutaneous ports or pumps.
• Optimization of site of injection/drug delivery based on the pain distribution.

## 8 Neuraxial safety

It is appreciated that local anesthetics can produce DRG neurolysis, and morphine can induce meningeally-derived granulomas (92–95). These phenomena have been shown to evolve in a *concentration* dependent fashion, which is compounded by the dilemma of maldistribution. It is of great importance that appropriate safety be determined in validated preclinical models before it is moved to the human platform. Variables impacting safety include the molecule itself, its concentration (vs. total dose where volume of delivery of a given concentration is increased) and its formulation constituents (e.g., adjuvants). This issue of concentration is an important consideration in the determination of safety. Increases in concentration above those studied must be considered as literally a novel therapeutic and its safety must be assessed. This criteria for clinical neuraxial use is recognized by many pain and anesthesia journals as a minimum criteria for publishing related results (96). The limited local pericatheter redistribution of intrathecal injectate, in view of the high solute concentrations employed in preclinical and clinical studies (97–99) with the relatively limited redistributional forces, leads to high persistent local peri-catheter solute concentrations proximal to the delivery site and accounts for many issues of local toxicity (see below) (69, 100, 101).

### 8.1 Related research topics

• Considerations of epidural or intrathecal therapeutics on local (DRG, root, Meninges and Parenchyma neuraxial toxicity in preclinical models.
• Role of concentrations vs. total local doses
• Patient associated pathology after neuraxial delivery.

The above brief overview reflects nonexclusively the primary areas of interest of the respective editors that this topical specialty seeks to address. We invite basic clinical and preclinical research and reviews with a focus on issues pertinent to neuraxial delivery of therapeutics in general and pain in particular. We point to a previous overview of this subject matter that was previously published in our journal (20).

## Data Availability

The original contributions presented in the study are included in the article/Supplementary Material, further inquiries can be directed to the corresponding author.
